# Non-contrast CT at comparable dose to an abdominal radiograph in patients with acute renal colic; impact of iterative reconstruction on image quality and diagnostic performance

**DOI:** 10.1007/s13244-014-0310-z

**Published:** 2014-02-07

**Authors:** P. D. McLaughlin, K. P. Murphy, S. A. Hayes, K. Carey, J. Sammon, L. Crush, F. O’Neill, B. Normoyle, A. M. McGarrigle, J. E. Barry, M. M. Maher

**Affiliations:** 1 Department of Radiology, Cork University Hospital, Wilton, Cork, Ireland; 2 Department of Radiology, University College Cork, Cork, Ireland; 3 Department of Radiology, Memorial Sloan-Kettering Cancer Center, New York, NY 10075 USA; 4 Department of Medical Physics, Cork University Hospital, Cork, Ireland

**Keywords:** Iterative reconstruction, Computed tomography, Renal colic, Nephrolithiasis, Ureterolithiasis, Radiation dose

## Abstract

**Objectives:**

The aim was to assess the performance of low-dose non-contrast CT of the urinary tract (LD-CT) acquired at radiation exposures close to that of abdominal radiography using adaptive statistical iterative reconstruction (ASiR).

**Methods:**

Thirty-three patients with clinically suspected renal colic were prospectively included. Conventional dose (CD-CT) and LD-CT data sets were contemporaneously acquired. LD-CT images were reconstructed with 40 %, 70 % and 90 % ASiR. Image quality was subjectively and objectively measured. Images were also clinically interpreted.

**Results:**

Mean ED was 0.48 ± 0.07 mSv for LD-CT compared with 4.43 ± 3.14 mSv for CD-CT. Increasing the percentage ASiR resulted in a step-wise reduction in mean objective noise (*p* < 0.001 for all comparisons). Seventy % ASiR LD-CT images had higher diagnostic acceptability and spatial resolution than 90 % ASiR LD-CT images (*p* < 0.001). Twenty-seven calculi (diameter = 5.5 ± 1.7 mm), including all ureteric stones, were correctly identified using 70 % ASiR LD-CT with two false positives and 16 false negatives (diameter = 2.3 ± 0.7 mm) equating to a sensitivity and specificity of 72 % and 94 %. Seventy % ASiR LD-CT had a sensitivity and specificity of 87 % and 100 % for detection of calculi >3 mm.

**Conclusion:**

Reconstruction of LD-CT images with 70 % ASiR resulted in superior image quality than FBP, 40 % ASIR and 90 % ASIR. LD-CT with ASIR demonstrates high sensitivity and specificity for detection of calculi >3 mm.

***Teaching Points*:**

• *Low-dose CT studies for urinary calculus detection were performed with a mean dose of 0.48 ± 0.07 mSv*

• *Low-dose CT with 70 % ASiR detected calculi >3 mm with a sensitivity and specificity of 87 % and 100 %*

• *Reconstruction with 70 % ASiR was superior to filtered back projection, 40 % ASiR and 90 % ASiR images*

## Introduction

Since its first description by Smith et al. in 1995, non-contrast CT of the urinary tract has become the imaging investigation of choice in patients with acute renal colic [1–3] because of its high sensitivity (95-97 %) and specificity (96-100 %) [2–5] for urinary tract calculi detection plus its high negative predictive value for urinary tract calculi. In addition, its accurate determination of the size and location of obstructing calculi allows clinicians to predict the likelihood of spontaneous passage [6, 7].

The necessity for radiation dose reduction is well recognised in this patient population as patients may present with their first episode of renal colic at a young age and recurrence in subsequent years is reported in 25-50 % of cases [8–10]. Many studies have examined the diagnostic efficacy of low-dose CT in the setting of renal colic and a variety of protocols have been described that result in effective dose reductions to as low as 0.5 to 3.5 mSv, achieved predominantly by reducing tube-time-current product values to as low as 7 mAs at a beam energy of 120 kV [11–21]. Low-dose CT is consistently associated with an increase in image noise but successes in dose reduction in the setting of renal colic have been aided by the inherent high contrast of renal calculi against the relatively low-density soft tissues surrounding the urinary tract.

Methods of iterative image reconstruction have been shown to successfully reduce image noise in conventional abdomino-pelvic CT and to date have facilitated dose reductions of between 20 and 60 % in a variety of phantom [22–26], in vivo adult [27–30] and in vivo paediatric studies [31].

Iterative reconstruction may be particularly useful in low-dose dose CT of the urinary tract where image noise is typically high. As intravenous contrast is rarely given in these studies, there are reduced inherent contrast differences between different abdominal organs, which is further compromised in low-dose CT images with increased image noise. Kulkarni et al. [32] have utilised adaptive statistical reconstruction in evaluating reduced dose non-contrast CT in patients with urolithiasis attending the ‘stone clinic’. The low-dose studies in this study had an average estimated dose of 1.8 mGy compared to 9.9 mGy for conventional dose studies. Vardhanabhuti et al. [33] have examined the benefits of iterative reconstruction in standard dose non-contrast CT for ureteric calculi detection. Our study is the first to investigate the potential benefits of iterative reconstruction for improving image quality and improving diagnostic capability in CT of the urinary tract, performed with a protocol designed to reduce the radiation dose to a level close to that of plain abdominal radiography (approximately 0.7 mSv at our institution) in the setting of suspected acute renal colic in emergency department patients.

Therefore we designed a prospective study involving the contemporaneous acquisition of conventional dose (CD-CT) (∼4 mSv) and low-dose non-contrast CT of the urinary tract (LD-CT) (∼0.5 to 0.7 mSv) with the following aims:

1. To determine the diagnostic performance of low-dose non-contrast CT of the urinary tract incorporating iterative reconstruction in the assessment of patients with suspected acute ureteric colic.
2. A secondary aim was to assess the impact of increasing increments of adaptive statistical iterative reconstruction (ASiR) on the image quality of these LD-CT images.
3. To determine the optimal percentage of iterative reconstruction for reconstructing LD-CT images by assessing changes in image quality when 40 % adaptive statistical iterative reconstruction (ASiR-40), 70 % adaptive statistical iterative reconstruction (ASiR-70) and 90 % adaptive statistical iterative reconstruction (ASiR-90) are used compared with traditional filtered back reconstruction (FBP).

## Materials and methods

Following approval by the institutional Research Ethics Committee, 33 consecutive symptomatic patients referred for CT from the emergency department with clinically suspected renal and/or ureteric calculi were prospectively included at a single centre. Exclusion criteria included patients in the paediatric age range, those who needed IV contrast and patients who had a metallic prosthesis. Written informed consent was obtained in each case by one of the investigators (SH). Patients who were scanned out of normal operational hours and when informed consent was not possible to obtain were also excluded. Data collection was planned before the index tests and reference standard were conducted consistent with a prospective study. Each patient had their weight and height measured using a digital device (Seca electronic measuring station, model 763, Seca Medical, Hamburg, Germany), and their body mass index (BMI) was subsequently calculated.

### CT acquisition technique

CT images were acquired using a 64-slice multi-detector row CT scanner (General Electric Lightspeed VCT-XTe, GE Healthcare, GE Medical Systems, Milwaukee, WI). In this study, all patients consented to have two non-contrast CT scans of the urinary tract performed instead of a single study; both CT scans covered an identical anatomic area extending from the lung bases to the pubic symphysis without intravenous contrast. The first CT protocol, however, was designed to impart an effective dose of 80-90 % of the effective dose of the standard departmental non-contrast CT scan of the urinary tract. This was known as the conventional dose CT scan (CD-CT) and served as the reference standard in this investigation. This CD-CT scan was followed immediately by a second low-dose non-contrast CT scan of the urinary tract (LD-CT) with a protocol designed to impart a radiation exposure of 10-20 % of the conventional CT scan. Using this strategy, the image quality and diagnostic yield of the LD-CT could be compared to that of the CD-CT scan and no patient would suffer additional radiation exposure (when exposure from the LD-CT and CD-CT were combined) as a result of recruitment into the study. The CD-CT protocol used a tube voltage of 120 kV, collimation of 0.625 mm, rotation time of 0.8 s and z-axis automated tube current modulation with minimum and maximum tube current thresholds set at 50 and 150 mA and a noise index of 52.0. The standard departmental protocol utilises the same collimation, rotation time and tube voltage, but has a tube current range of 60-170 mA and noise index of 45. The LD-CT protocol employed a tube voltage of 100 kV, collimation of 0.625 mm, rotation time of 0.5 s and fixed tube-time-current product of 35-40 mAs. The fixed tube-time-current product technique was preferred over the use of z-axis automated tube current modulation (ATCM) to ensure that radiation exposure remained below that of plain radiography regardless of body habitus; the use of ATCM would result in higher radiation exposures with increasing body mass index and thus the final radiation exposure would be less predicatable. Single medio-lateral and antero-posterior scout views were obtained prior to both helical acquisitions. The CD-CT images were reported in routine clinical fashion and LD-CT images were used for research purposes alone, i.e. they were not used to guide clinical decision-making at the time of scanning.

### Effective dose measurements

Dose length product (DLP) values were recorded from the dose report for CD-CT and LD-CT protocols. The imaging performance and assessment in the CT patient dosimetry calculator (ImPACT version 0.99×, London, England) were used to calculate the effective dose (ED) in all cases. For the purpose of comparing the effective dose between the CD-CT and LD-CT protocols, the radiation exposure resultant from the topogram was excluded from calculations. Calibration of the CT unit was performed once per week in accordance with the manufacturer’s instructions.

### CT image reconstruction

Raw data sets from both the CD-CT and LD-CT protocols were reconstructed at the scanner console using standard FBP and 40 % ASiR (60 % FBP) algorithms, the standard reconstruction technique employed in the department. The LD-CT raw data sets were additionally reconstructed with 70 % ASiR (30 % FBP) and 90 % ASiR (10 % FBP) algorithms in an attempt to further reduce image noise. Manufacturer guidelines (and default settings on the CT scanner) indicated that ASiR-40 is the optimum reconstruction method for conventional dose images; hence the CD images were not reconstructed using higher levels of ASiR. All images were reconstructed to a slice thickness of 2 mm (average), 5 mm (average) and 5 mm (maximum intensity projection, MIP) for the purposes of subjective and objective analysis of image quality.

### Quantitative analysis of image noise

Spherical regions of interest (ROIs) (diameter, 10 mm; volume, 519 mm^3^) were placed in the following nine anatomic structures by a single reader (KC, 1 year experience): liver at the diaphragm, liver at the porta hepatis, renal cortex at the renal hilum, psoas muscle at the iliac crest, bladder, gluteus maximus at the level of the top of the acetabulum, buttock fat, ischiorectal fossa fat and intraperitoneal fat in the left upper quadrant. Background noise was determined by placing the ROI in the air outside the patient’s body. In each structure, efforts were made to place the ROI in as homogeneous an area as possible, away from blood vessels, fat planes, etc. Mean attenuation in HU and standard deviation of the mean attenuation in the ROI were recorded on CD-CT and LD-CT images reconstructed with FBP, ASiR-40, ASiR-70 and ASiR-90 including 2 mm, 5 mm and 5 mm MIP reformats. The standard deviation of the mean attenuation in the ROI served as an objective measure of noise [34]. The signal-to-noise ratio (SNR) within each ROI was calculated by dividing the mean HU by its standard deviation [35]. It was not possible to accurately place the ROIs within the required anatomical structures in two patients (both with BMI > 40) because of severe degradation of image quality in the LD-CT series; therefore quantitative analysis of image noise was performed in 31 of 33 patients equating to a total of 11,160 data points for analysis (31 patients; 4 LD reconstructions; 3 reformat types; 10 ROIs; 3 values per ROI). Objective noise indices were statistically compared by deriving the mean objective noise (SD of HU within ROI) at the ten measured levels for each data set.

### Subjective image quality

All reconstructed CT images were reviewed in the Digital Imaging and Communications in Medicine (DICOM) format on a commercial workstation (Advantage Workstation VolumeShare 2, version 4.4, GE Medical Systems, Milwaukee, WI) using soft-tissue window settings only (window width, 400 HU; window level, 40 HU). The subjective image quality parameters and grading system were adapted from the European Guidelines on Quality Criteria for CT document [36] and were selected on the basis of findings of previous studies [28, 37]. One of the observers (MMM, 17 years' experience) was familiar with these methods of assessment, having successfully used them previously [38–40], and trained the other reader (PMcL, 5 years' experience) prior to analysis using a training set of five standard CTs.

Diagnostic acceptability, subjective image noise and spatial resolution were subjectively scored in consensus (MMM, PMcL) using a ten-point scale at five anatomical levels: right hemi-diaphragm, porta hepatis, right renal hilum, iliac crest and roof of the acetabulum. In assessing spatial resolution, the edge clarity of solid viscera and skeletal muscles at the relevant levels were scrutinised by the readers. The presence and impact of streak artefact was also scored at each anatomical level using a 3-point scheme (0, no streak artefact; 1, streak artefact present but not interfering with image interpretation; 2, streak artefact present and interfering with image interpretation). Contrast resolution was scored using a 10-point scale at three levels: liver, spleen and gluteus maximus. Qualitative indices were recorded on CD-CT and LD-CT images reconstructed with FBP, ASiR-40, ASiR-70 and ASiR-90 including 2 mm, 5 mm and 5 mm MIP reformats yielding 13,685 data points for analysis.

Diagnostic acceptability was graded as acceptable (score of 5), unacceptable (score of 1) or excellent (score of 10), respectively, if depiction of soft-tissue structures for diagnostic interpretation and degree of image degradation by beam-hardening artefacts was satisfactory, unsatisfactory, or considerably superior. Subjective image noise was graded according to the extent of “graininess” or “mottle” present on CT images and was graded as acceptable (score of 5) if average graininess was seen with satisfactory depiction of small anatomic structures such as the blood vessels and interface between structures of variable attenuation, unacceptably high (score of 10) if graininess interfered with depiction of these structures and extremely low (score of 1) where there was minimal or no appreciable mottle. With regard to contrast resolution, a score of ten represented superior contrast depiction between different soft tissues, a score of one indicated the most inferior contrast and five indicated acceptable contrast.

### Detection of urinary calculi

Results of the qualitative analysis were compiled and the optimal LD-CT data set (5 mm ASiR-70) was read in consensus by two radiologists (JEB, 22 years' experience; PMcL, 5 years' experience). Access to the clinical request details and CD-CT images was withheld and first, as a measure of quality and ease of interpretation, the conspicuity of the course of the ureters was scored on a scale of 1–4 (1 = impossible to trace; 2 = difficult to trace; 3 = possible to trace; 4 = easily traced). The number, location, size and density of each calculus were documented. The presence or absence of other urinary tract imaging findings such as renal enlargement, pyeloureteral dilation, perirenal/periureteral stranding and renal tract obstruction were also recorded. Any additional, extra-urinary tract findings including incidental findings and those relevant to a presentation of acute abdominal pain were also recorded as per standard clinical practice.

The optimal CD-CT data set (5 mm ASiR-40) (40 % ASiR and 60 % FBP), which is the default reconstruction algorithm as per the CT manufacturer’s guidelines, was chosen as the gold standard based on superiority performance on image quality analysis. The 5-mm CD-CT ASiR-40 images were read in consensus immediately following review of the LD-CT images (JEB, PMcL). The number, location, size and density of missed calculi were documented. The relative conspicuity of missed calculi between the 5-mm CD-CT ASiR-40 and the 5-mm LD-CT images reconstructed with FBP, ASiR-70 and ASiR-90 MIP was scored on a 6-point scale (0 = not seen; 1 = much worse; 2 = slightly worse; 3 = equal; 4 = better; 5 = excellent). Discrepant extra-urinary tract findings were scored according to the ACR RADPEER scoring system [41].

### Statistical analysis

All statistical tests were performed with a commercially available medical statistical package (PASW version 20, SPSS Inc., Chicago, IL, USA) (PMcL). As image analysis was performed in consensus between readers; we did not perform a κ test of inter-observer agreement. The Wilcoxon signed rank test was used for statistical analysis to compare the qualitative parameters (diagnostic acceptability, image noise, streak artefact, spatial resolution and contrast resolution), and normally distributed quantitative indices were compared using a paired t-test. Correlation of parametric and non-parametric variables was performed using Pearson and Spearman’s tests, respectively. A difference with a *p* value of <0.05 was considered statistically significant.

## Results

Thirty-three patients, 16 females and 17 males with a mean age of 45.2 ± 16.3 years (age range, 16–74 years) and mean BMI of 26.9 ± 6.7 kg/m^2^ (BMI range, 18.2-47.4 kg/m^2^), were included in this study. Patients were recruited over a 3-month period between September and December 2010.

### Effective dose

Mean DLP for LD-CT (34.18 ± 5.3 mgy.cm) was significantly lower than the mean DLP for CD-CT (272.17 ± 190.99 mgy.cm) (*p* < 0.001). Mean ED, as individually calculated using ImpaCT dosimetry software, was 0.48 ± 0.07 mSv for LD-CT compared with 4.43 ± 3.14 mSv for CD-CT. The LD-CT protocol resulted in a DLP of 82.9 ± 8.0 % less than the CD-CT protocol. A statistically significant increase in DLP (*p* < 0.001) was encountered with increasing BMI when the CD-CT protocol was used, but as expected, there was no significant increase in DLP (*p* = 0.279) with increasing BMI when the fixed-tube-current LD-CT protocol was employed. As a result, there was a greater percentage reduction in DLP with the fixed tube current LD-CT protocol with increasing patient BMI (Spearman’s correlation 0.808, *p* < 0.001).

### Image quality: objective analysis

**Fig. 1:**
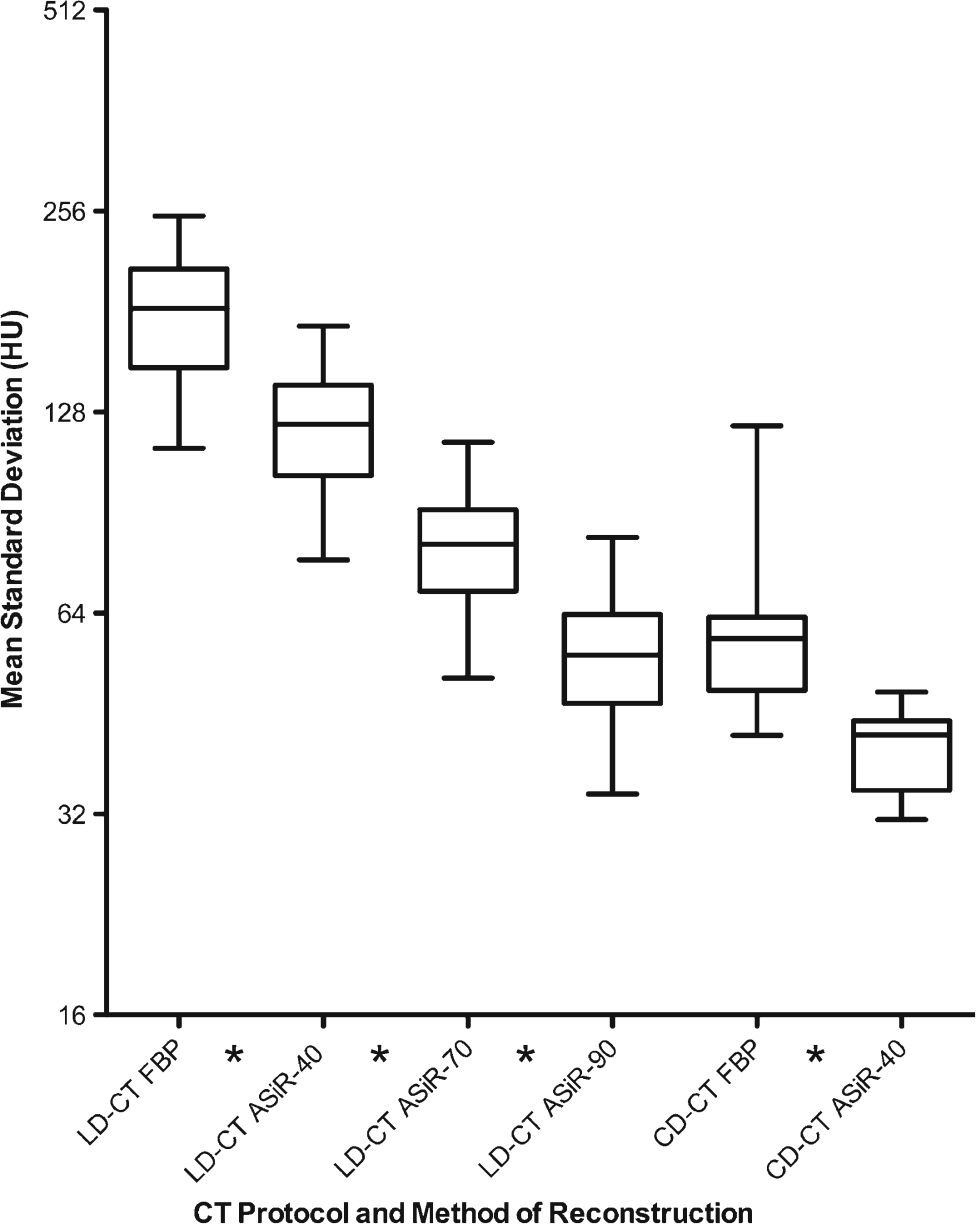
Changes in mean objective noise (mean standard deviation of HU measured in 10 anatomical levels) encountered when 5-mm LD-CT images were reconstructed with FBP, and ASiR-40, ASiR-70, ASiR-90 and CD-CT images were reconstructed with FBP and ASiR-40 algorithms. (*Star symbol* denotes statistically significant difference, *p* < 0.05)

Objective noise was significantly lower on 5-mm images than on 2-mm and 5-mm MIP images for all methods of reconstruction (FBP, ASiR-40, ASiR-70 and ASiR-90) (*p* < 0.001). Objective noise on the 5-mm CD-CT images was significantly lower when images were reconstructed using ASiR-40 compared with FBP (*p* < 0.000). Reconstructing the 5-mm LD-CT data sets with increasing percentages of ASiR resulted in a statistically significant progressive reduction in mean objective noise over all ten measured anatomic levels (*p* < 0.000 for all comparisons) (Fig. 1), and no significant difference in mean objective noise was found when 5-mm CD-CT images, reconstructed with FBP, were compared with the 5-mm LD-CT images reconstructed with ASiR-90 (*p* = 0.453) (Fig. 1).

There was a statistically significant correlation between patient BMI and mean objective image noise on all LD-CT data sets (fixed tube current) (Pearson's correlation 0.301, *p* = 0.01). Conversely, no statistically significant correlation was found between patient BMI and mean objective image noise on all CD-CT images (ATCM) (Pearson's correlation −0.097, *p* = 0.459). This finding reflects the use of ATCM on the CD-CT protocol and fixed tube current technique with the LD-CT protocol.

### Image quality: subjective analysis

#### CD-CT protocol

Median diagnostic acceptability, spatial resolution and contrast resolution scores were significantly higher and subjective noise scores were significantly lower on 5-mm images compared with 2-mm and 5-mm MIP images for both reconstruction algorithms (FBP, ASiR-40) (*p* < 0.001 for all comparisons). Both FBP and ASIR-40 5-mm CD-CT images had above average to excellent diagnostic acceptability, spatial and contrast resolution, but when images generated from the two algorithms were compared significantly higher diagnostic acceptability, spatial resolution and contrast resolution scores were achieved by the ASiR-40 images (*p* < 0.001 for all comparisons) when compared to those reconstructed with FBP. CD-CT FBP and CD-CT ASiR-40 images had equal median streak artefact scores (*p* = 1).

#### LD-CT protocol

Median diagnostic acceptability, spatial resolution and contrast resolution scores were significantly higher on 5-mm images than on 2-mm and 5-mm MIP images for all reconstruction algorithms (FBP, ASiR-40, ASiR-70 and ASiR-90) (*p* < 0.001 for all comparisons; Appendix 1). Similarly subjective noise scores were significantly lower on 5-mm images than on 2-mm and 5-mm MIP images for all methods of reconstruction (*p* < 0.001 for all comparisons, Appendix 1). Streak artefact scores were lower on the 5-mm MIP images when compared with the 2-mm and 5-mm reconstructions, but this trend only reached statistical significance on the series reconstructed with FBP (Wilcoxon signed rank test, *p* = 0.046).

**Fig. 2:**
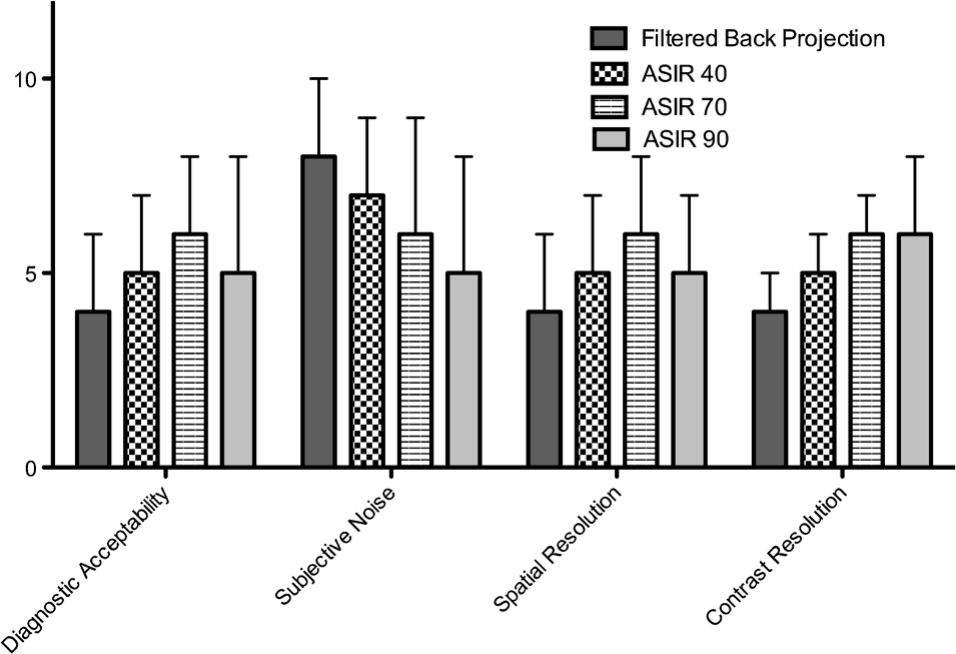
Changes in subjective image quality parameters (median score as measured at 5 anatomical levels) encountered when 5-mm LD-CT images were reconstructed with FBP, ASiR-40, ASiR-70 and ASiR-90. (All differences were statistically significant except comparison of contrast resolution scores between ASiR-70 and ASiR-90 images)

There was a progressive improvement in subjective image quality indices when ASiR-40 and ASiR-70 were applied to the LD-CT images (*p* < 0.001 for all comparisons). Increasing the percentage of ASiR from 70 to 90 % resulted in a significant decrease in subjective noise but also importantly resulted in a significant decrease in the diagnostic acceptability and spatial resolution score due to a subjective “over-smoothening” of tissue interfaces (*p* < 0.001). There was no significant difference in streak artefact or contrast resolution scores between ASiR-70 and ASiR-90; therefore, the images reconstructed at a slice thickness of 5 mm with 70 % ASIR were chosen as the LD-CT data set with optimal image quality and were chosen for the clinical analysis of stone conspicuity (Fig. 2).

**Fig. 3:**
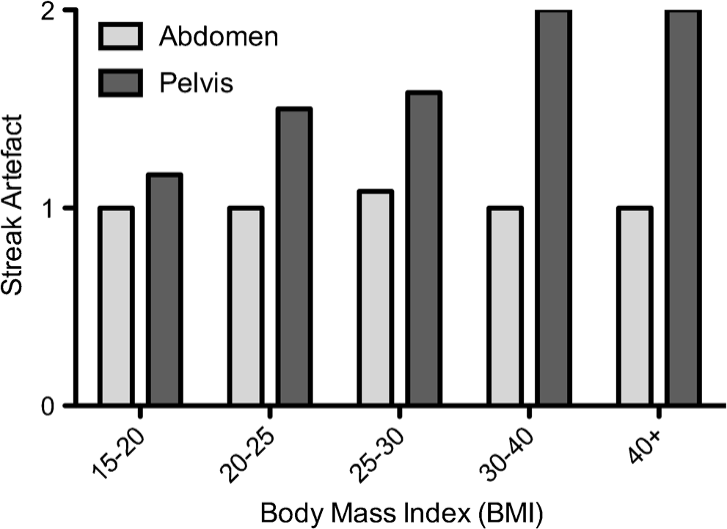
Changes in streak artefact score in the abdomen (light grey) and pelvic (dark grey) stations in patients with increasing BMI. 0, no streak artefact; 1, streak artefact present but not interfering with image interpretation; 2 streak artefact present and interfering with image interpretation

Streak artefact scores were uniformly one or less on the CD-CT images, which employed ATCM, but streak artefact scores of 2 (interfering with image interpretation) were encountered on the LD-CT images particularly in the pelvis. Pelvic streak artefact, interfering with image interpretation, on the LD-CT images was seen in all patients with a BMI of >30 (Fig. 3).

### Detection of urinary calculi

The conspicuity of the course of the ureters was scored, as a measure of examination quality and ease of interpretation, for each LD-CT ASiR-70 data set as well as the CD-CT ASiR-40 images. On the LD-CT ASiR-70 images, the right ureter was impossible to fully trace along its length in 15 patients, was difficult in 14 patients and was scored as possible only in 3 cases. One patient had a previous right nephro-ureterectomy. The left ureter was impossible to fully trace along its length in 13 patients, was difficult in 16 patients and was scored as possible in 4 cases. On the CD-CT ASiR-40 images, however, only 5 of these 65 ureters were deemed difficult or impossible to trace with the remaining 60 being possible to trace or easily traced.

**Fig. 4:**
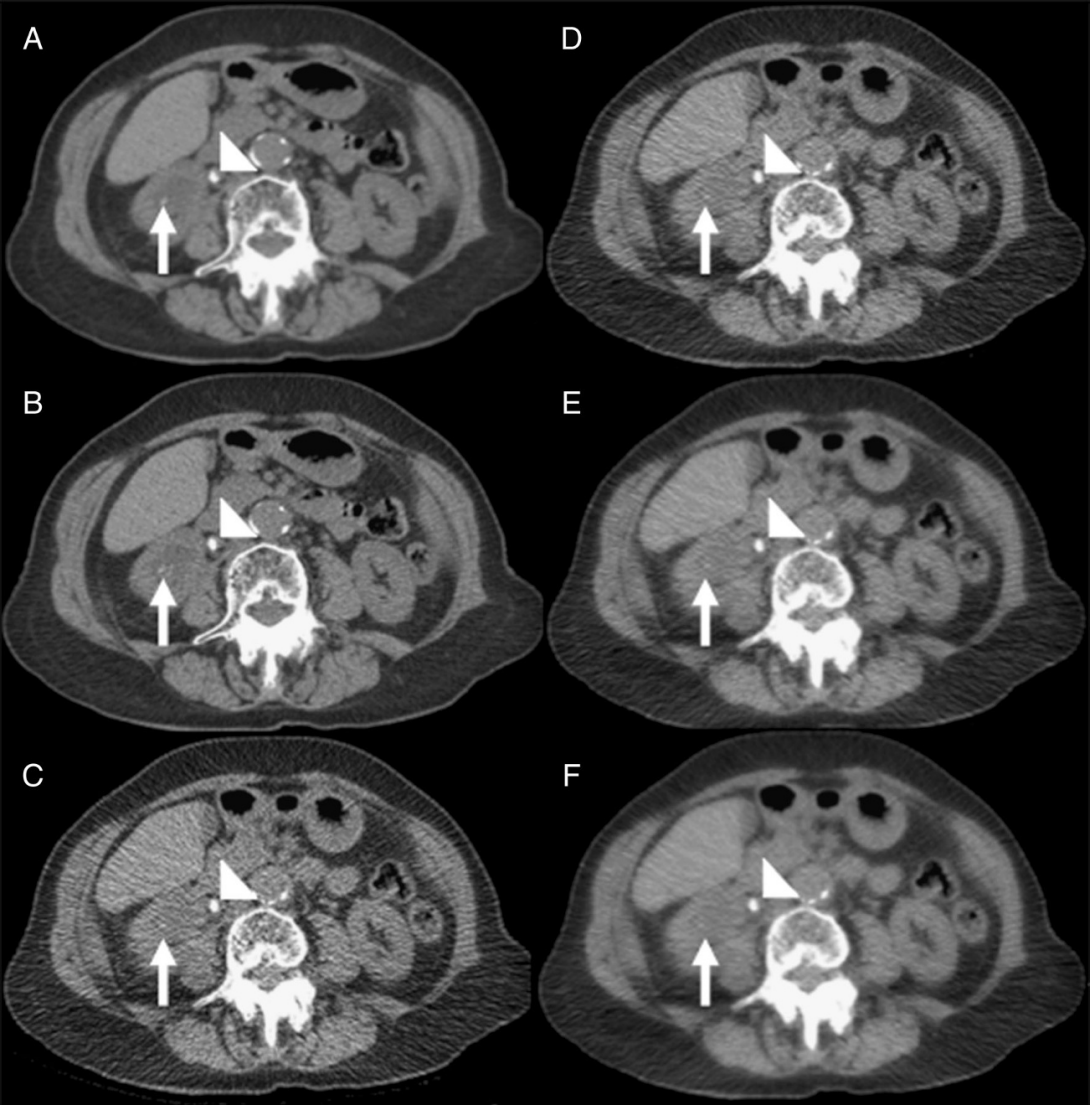
A 48-year-old female patient (BMI = 25.6) with acute right ureteric colic. **a and b** Axial conventional dose (5.1 mSv) non-contrast CT image reconstructed with 40 % ASiR (**a**) and 100 % FBP (**b**) showing an 8-mm right ureteric calculus (*arrowhead*) and an additional 2-mm renal calculus in the right lower pole (*arrow*) that was not prospectively detected on the low-dose images. **c**-**f** Axial low-dose (0.56 mSv) non-contrast CT image reconstructed with 100 % FBP (**c**), 40 % ASiR (**d**), 70 % ASiR (**e**) and 90 % ASiR (**f**) showing the obstructing 8-mm calculus in the proximal right ureter (*arrowhead*). The location of the smaller 2.1-mm calculus is also demonstrated (*arrow*). Note the ‘over smoothening’ of the soft tissues on the 90 % ASiR image compared to the other low-dose reconstructions

**Fig. 5:**
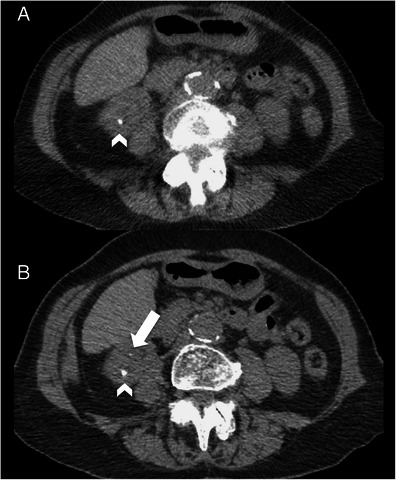
A 53-year-old female patient (BMI = 26.3) with acute left ureteric colic. **a** Axial low-dose (0.43 mSv) 2-mm non-contrast CT image reconstructed with 70 % ASiR showing a 5-mm calculus in the lower pole of the right kidney (*arrowhead*). **b** Axial conventional dose (5 mSv) 2-mm non-contrast CT image reconstructed with 40 % ASiR showing an additional 1.7-mm renal calculus in the right lower pole (*arrow*)

Twenty-seven calculi were identified in 15 patients on the LD-CT ASiR-70 images with a mean diameter of 5.5 ± 1.7 mm. A total of 16 additional calculi were found in 11 patients when the CD-CT ASiR-40 images were reviewed, and there were two false-positive calculi (same patient) encountered on the LD-CT ASiR-70 images (Figs. 4 and 5). The 16 calculi that were missed on the LD-CT ASiR-70 images were significantly smaller (mean diameter 2.3 ± 0.7 mm) than those 27 identified on the LD-CT ASiR-70 images (*p* < 0.001), and all missed calculi were located in the kidneys. All ureteric calculi were successfully identified on the LD-CT ASiR-70 images (total = 9, mean diameter 6.6 ± 1.4 mm). The BMI of patients in whom calculi were missed did not significantly differ from those in whom calculi were correctly identified (*p* = 0.618). Signs of pyeloureteral dilation, renal enlargement and urinary tract obstruction were correctly identified in seven out of seven patients on the LD-CT ASiR-70 images. Peri-renal stranding was correctly identified in five patients but was missed in one patient when LD-CT ASiR-70 images were compared with the CD-CT ASiR-40 data set.

### Sensitivity and specificity

Of the 33 patients investigated, 18 had urinary calculi. Thirteen patients had urinary calculi correctly found on the LD-CT ASiR-70 images (true positive) and five patients had urinary calculi missed on the LD-CT ASiR-70 images (false negative). Urinary calculi were correctly excluded in 15 patients (true negative) and there was one false-positive case encountered on the LD-CT ASiR-70 images involving two calculi in the same patient. Therefore, the sensitivity and specificity of the LD-CT ASiR-70 images for detecting all urinary tract calculi in this group of patients are 72 % and 94 %, respectively, with a positive predictive value (PPV) of 93 % and negative predictive value (NPV) of 75 %. When only calculi 3 mm or greater in diameter are considered, the sensitivity and specificity rise to 87 % and 100 %, respectively. In this subgroup the PPV is 100 % and the NPV is 91 %.

### Detection of extra-urinary tract findings

**Fig. 6:**
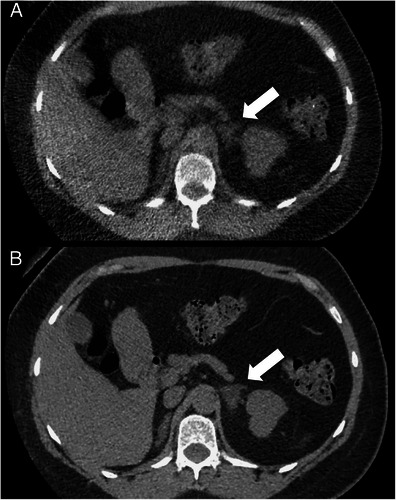
A 40-year-old female patient (BMI = 29.8) with suspected renal calculi. **a** Axial low-dose (0.45-mSv) 2-mm non-contrast CT image reconstructed with 70 % ASiR and **b** axial conventional dose (7 mSv) 2-mm non-contrast CT image reconstructed with 40 % ASiR showing a 3.2-cm low-density left adrenal mass consistent with a benign adenoma (*arrow*). This left adrenal mass was missed when viewed on the low-dose non-contrast CT images

**Fig. 7:**
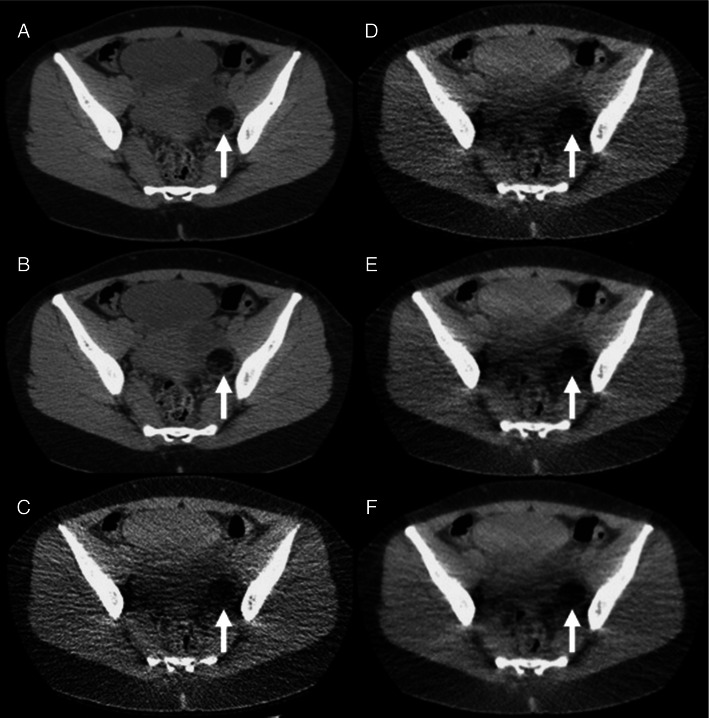
A 29-year-old female patient (BMI = 20.8) with haematuria. **a and b** Axial conventional dose (3.6-mSv) 5-mm non-contrast CT images reconstructed with 40 % ASiR (**a**) and 100 % FBP (**b**) showing a 5.4-cm low-density left adnexal mass consistent with a dermoid cyst (*arrow*). This left adnexal mass was missed when viewed on the low-dose non-contrast CT images. **c**-**f** Axial low-dose (0.48-mSv) 5-mm non-contrast CT images reconstructed with 100 % FBP (**c**), 40 % ASiR (**d**), 70 % ASiR (**e**) and 90 % ASiR (**f**) showing the inferior detail of the left adnexal lesion relative to the conventional dose images (*arrow*). Again note the ‘over smoothening’ of the soft tissues on the 90 % ASiR image compared to the other low-dose reconstructions

Many important and clinically significant extra-urinary tract findings were missed while interpreting the LD-CT ASiR-70 images (Table 1). These included two cases of acute appendicitis, two adrenal adenomas (Fig. 6) and one 3-cm ovarian dermoid cyst (Fig. 7), which were graded as RADPEER four discrepancies after peer review (discrepancy in interpretation/should be made almost all of the time). Extra-urinary tract findings that were correctly identified included four cases of colonic diverticulosis (non-complicated), consolidation at the left lung base in one patient and various hyperdensities (see Table 1). The correctly identified findings all had subjectively high lesion-to-background contrast due to calcific or gaseous interfaces with the background soft tissue. The other LD-CT reconstructions (FBP, ASiR-40 and ASiR-90) were found to have similar sensitivity in detection of extra-urinary tract findings.

**Table 1:** Summary of the extra-urinary tract findings identified and missed on the LD-CT ASiR 70 images

Correctly found on LD-CT ASIR 70 images	Number of patients	Findings missed on LD-CT ASIR 70 images	Number of patients
Diverticulosis	4	*RADPEER 4b*	
Left lung consolidation	1	Appendicitis	2
*Hyperdensities*:		Ovarian dermoid	1
Vascular calcification	5		
Gallstones	3	*RADPEER 4a*	
Hepatic granuloma	4	Adrenal adenoma	2
Calcified mesenteric nodes	2		
Pancreatic calcification	1	*RADPEER 3a*	
Prostate brachytherapy	1	Hepatic steatosis	2
Rectal sutures	1	Gallstones	2
Prostate calcification	1	Hepatic cyst	1
Calcified uterine fibroid	1	Enlarged uterus/cervix	2
		Previous colectomy	1

Discrepant extra-urinary tract findings are scored according to the ACR RADPEER scoring system [37]

## Discussion

Many previous studies have examined the diagnostic efficacy of low-dose CT in the setting of renal colic by employing a wide range of phantom [42–44], software-based [45, 46], cadaveric [47] and in-vivo [11–21] study designs. Common to all of these previous studies is an examination of the influence of increased image noise on the detection of urinary tract calculi at low-dose computed tomography. To the best of our knowledge, no study to date has examined the effect of recently available iterative reconstruction techniques, developed with the aim of reducing image noise in reduced-dose CT images, on image quality and diagnostic performance of non-contrast CT in the setting of acute renal colic. A study by Kulkarni et al. [32] reported on an investigation of the effectiveness of iterative reconstruction in unenhanced CT of the urinary tract. However, this study looked at the effectiveness of IR in CT studies performed at much higher radiation doses than in the current study and was performed in patients with urolithiasis attending the urology ‘stone clinic’ as opposed to patients attending the emergency department with acute pain from clinically suspected ureteric colic. In comparison, our low-dose CT protocol had a much lower radiation dose, comparable to plain radiography, and these CT studies were performed in the clinical setting of acute abdominal pain and suspected ureteric colic.

We found significant improvements in subjective and objective image quality indices when low-dose CT images were reconstructed with a commercially available iterative reconstruction technique, namely adaptive statistical iterative reconstruction (GE Healthcare, GE Medical Systems, Milwaukee, WI) when compared with low-dose images reconstructed with filtered back projection. In addition, we also found that conventional dose CT images (mean effective dose 4.4 mSv) reconstructed with 40 % ASiR had significantly higher image quality indices than when reconstructed with FBP. CT images acquired using our low-dose protocol had high subjective and objective image noise, and consequently we investigated the potential benefits of higher percentages of iterative reconstruction on image quality and diagnostic acceptability and yield. We found a statistically significant reduction in objective image noise with increasing percentages of ASiR. The effectiveness of iterative reconstruction in removing noise is highlighted by the finding that there was no significant difference in objective image noise between the LD-CT ASiR-90 images and the CD-CT images, reconstructed with FBP despite an 82 % mean reduction in DLP. Subjective image quality indices also improved with increasing percentages of ASiR but the relationship was not as linear as with objective noise scores. For LD-CT images, there was a statistically significant reduction in diagnostic acceptability and spatial resolution scores when images were reconstructed with 90 % ASiR because of a subjective “over-smoothening” of the tissue interfaces suggesting that the 70 % ASiR algorithm is optimal for the clinical interpretation of LD-CT images with dose reductions of the above-described order. It should be noted that despite an improvement in image quality with the ASiR-70 algorithm, median scores for diagnostic acceptability, subjective noise, spatial resolution and contrast resolution at this level of dose remained low. Streak artefact frequently interfered with image interpretation in the pelvis in all LD-CT images because of the low photon fluence and also the fixed tube current time product; increasing BMI had a deleterious effect on this and significant streak artefact in the pelvis was seen in 100 % of patients with BMI of over 30 kg/m^2^. It must be highlighted in considering image quality data, however, that the level of dose reduction achieved in this study population is extreme when one considers that the mean effective dose was 0.48 mSv with a standard deviation of 0.07, confirming that this dose level was achieved across the whole study population, which had a mean BMI of 26.9 and a range of 18.2–47.7 kg/m^2^.

In relation to the detection of urinary tract calculi, we subjectively found a large proportion of the LD-CT ASiR-70 images difficult to interpret. This was reflected in the difficulty in tracing the ureters on the LD-CT images. In spite of this, however, sensitivity and specificity for detecting calculi greater than 3 mm in diameter with the LD-CT ASiR-70 images were 87 % and 100 %. In our study, however, when all urinary calculi were included, sensitivity and specificity when using the LD-CT ASiR-70 images were reduced to 72 % and 94 %, respectively. It is worth noting that all ureteric calculi were retrospectively detected on the ASiR-70 images. Important signs of pyeloureteral dilation, renal enlargement and urinary tract obstruction were correctly identified in seven out of seven patients on the LD-CT ASiR-70 images but peri-renal stranding was missed in one patient, likely reflecting the effect of increased image noise on contrast resolution. It is important again, however, to highlight that these CT images were acquired at a mean effective dose of 0.48 mSv, less than the mean effective dose of a plain abdominal radiograph at our institution (0.7 mSv). A previous study by Ege et al. from 2004 reported that only 57 of 111 stones (57 %) detected on CT were identified on plain radiography and that the sensitivity of plain abdominal radiography for stones less than 5 mm was only 37 % [48].

Clinically significant extra-urinary tract findings were missed on the LD-CT ASiR-70 images including two cases of acute appendicitis and one 3-cm ovarian dermoid. Larger series in the literature report a similar frequency (5-10 %) of alternate causes of flank pain and clinically significant findings in non-contrast-enhanced CTs of the urinary tract [49, 50] and failure to diagnose these clinically relevant conditions implies that the LD-CT protocol investigated in this study, even with the help of iterative reconstruction, could not be promoted as a replacement for conventional dose CT in the acute setting. Based on the findings of this study, however, it is likely that the LD-CT protocol, which is performed at a radiation exposure less than that of plain radiography (0.48 mSv), could be used in clinical situations, where follow-up CT or plain radiography is required to determine whether urinary tract calculi have passed or moved. With the development of newer reconstruction techniques, such as model-based iterative reconstruction, ultra-low-dose CT protocols at radiation doses utilised in this study could be utilised as a first-line investigation in patients with clinically suspected urinary calculi.

Our study is not the first in the literature to examine the diagnostic accuracy of LD-CT in the range of 0.5–0.7 mSv in the setting of urinary tract calculi. Kluner et al. [20] report a sensitivity of 86–96 and specificity of 97–100 in 2006 using 16-MDCT with 120 kVp, pitch 1.43 and a mean mAs of 6.9. A recognised limitation in this and other studies is the choice of gold standard investigation on which sensitivity and specificity values are based. It is clear that studies such as the aforementioned one by Kluner et al. in which the findings on the reduced dose CT protocol under investigation were compared with findings on urography, ureterography, endoscopy and/or clinical follow-up were limited with potential for inappropriately low false-negative cases and consequently a spurious increase in reported sensitivity [20]. The major strength of the current study is that the low-dose technique was directly compared with contemporaneous conventional dose CT images, and thus sensitivity and specificity values are much more robustly evaluated.

A possible limitation of the current study was that the CD-CT protocol used as our “gold standard” was designed to deliver a CT examination with a radiation exposure equivalent to our routine departmental non-contrast CT dose minus 10-20 %. This decision was ethically based as our group considered it inappropriate to subject the study group to additional radiation doses in a research setting. This strategy would appear further justified by the age profile of the study population (mean age 45 years). Our chosen gold standard therefore represents a compromise in the interests of the safety of recruited patients. It was very reassuring, however, that both subjective and objective analysis of the CD-CT images suggested uniformly excellent image quality and calculi as small as 1.5 mm were identified with ease; therefore, we believe that the choice of gold standard in our study ethically and practically represents the optimal comparison in living patients. It is also represents a much more robust gold standard when compared to other radiation dose studies where the gold standard CT examination comprises a higher dose CT examination performed months earlier. Another possible limitation of the study was our decision to use “fixed tube current” rather than automatic tube current modulation (ATCM) techniques for the purpose of acquiring the LD-CT images at an effective dose of less than 0.7 mSv. ATCM was employed for the CD-CT protocol. The decision not to employ ATCM for the LD-CT study was taken, as our aim was to assess the effectiveness of a protocol (aided by iterative reconstruction), which imparted a radiation exposure less than plain radiography in detecting urinary tract calculi. Detailed consideration of the protocol by our multidisciplinary team of technologists, radiologists and medical physicists concluded that for the entire study population encompassing all BMIs, the use of ATCM was likely to result in failure to achieve the goal of imparting an effective dose of less than 0.7 mSv, particularly in patients with higher BMIs. Our LD-CT protocol was successful in imparting the required dose (mean 0.48 mSv) across a range of BMIs. However, this protocol requires further modification to improve image quality especially in the pelvis.

In conclusion, LD ASiR-70 CT images (with 70 % iterative reconstruction) allowed the detection of calculi greater than 3 mm in size with a sensitivity and specificity of 87 % and 100 % at a mean ED (0.48 ± 0.07 mSv) comparable to a single abdominal radiograph. When calculi of all sizes are taken into account, the LD ASiR-70 images demonstrated a sensitivity and specificity of 72 % and 94 % because of inferior detection of sub-3-mm calculi. All ureteric calculi were detected on the LD ASiR-70 images. Unfortunately, clinically significant extra-urinary tract findings were missed on the LD-CT images. Utilisation of this protocol could not be advocated as a replacement for conventional dose CT imaging in the initial assessment of patients with renal colic. A genuine potential use would be in following up patients with known calculi to determine whether urinary tract calculi have passed or moved (Appendix 2).

## Appendixes

### Appendix 1

**Table 2:** Summary of subjective image quality analysis of 2-mm, 5-mm and 5-mm MIP LD-CT data sets

		FBP m(SD)	*P*-value	ASIR-40 m(SD)	*P*-value	ASIR-70 m(SD)	*P*-value	ASIR-90 m(SD)	*P*-value
Diagnostic acceptability	2 mm	3(2)	0.000^a^	4(2)	0.000^a^	5(2)	0.000^a^	5(2)	0.000^a^
	5 mm	4(2)	0.000^b^	5(2)	0.000^b^	6(2)	0.000^b^	5(2)	0.000^b^
	5 mm MIP	2(1)	0.001^c^	3(1)	0.002^c^	4(1)	0.026^c^	4(2)	0.143^c^
Subjective noise	2 mm	9(1)	0.000^a^	8(0)	0.000^a^	7(0)	0.000^a^	6(0)	0.000^a^
	5 mm	8(0)	0.000^b^	7(0)	0.000^b^	6(1)	0.000^b^	5(1)	0.000^b^
	5 mm MIP	9(1)	0.026^c^	8(1)	0.022^c^	7(1)	0.016^c^	6(1)	0.012^c^
Spatial resolution	2 mm	4(1)	0.000^a^	4(1)	0.000^a^	5(2)	0.000^a^	5(2)	0.000^a^
	5 mm	4(1)	0.000^b^	5(1)	0.000^b^	6(1)	0.000^b^	5(1)	0.000^b^
	5 mm MIP	3(1)	0.015^c^	4(1)	0.075^c^	5(1)	0.068^c^	5(1)	0.693^c^
Contrast resolution	2 mm	3(1)	0.000^a^	4(2)	0.000^a^	4(2)	0.000^a^	5(2)	0.000^a^
	5 mm	4(1)	0.000^b^	5(1)	0.000^b^	6(2)	0.000^b^	6(2)	0.000^b^
	5 mm MIP	2(1)	0.001^c^	3(1)	0.007^c^	4(1)	0.088^c^	5(1)	0.144^c^

### Appendix 2

**Table 3:** Summary of the 16 calculi that were missed in 11 patients on the LD-ASIR 70 images

	Size (mm)	Location	Obstructing (y/n)	Attenuation (HU)	LD-CT FBP (conspicuity)	BMI	DLP for LD-CT	DLP for CD-CT
1	2.1	Renal-left inter pole	n	303	0	35.6	46.23	496.84
2	1.5	Renal-left inter pole	n	183	0	35.6	46.23	496.84
3	2	Renal-left inter pole	n	212	0	35.6	46.23	496.84
4	1.3	Renal-left inter pole	n	139	0	35.6	46.23	496.84
5	3.5	Renal left lower pole	n	437	0	29.2	33.99	474.96
6	2.3	Renal-right lower pole	n	278	0	47.4	32.6	917.92
7	2.4	Renal-left inter pole	n	425	0	22.9	33.31	158.84
8	2.5	Renal-right inter pole	n	302	0	19.7	30.18	171.24
9	2.4	Renal-left inter pole	n	314	0	26.3	29.64	310.88
10	1.9	Renal-right lowr pole	n	201	0	18.2	32.5	129
11	2.3	Renal-left inter pole	n	215	0	28.8	33.77	424.34
12	3	Renal-right lower pole	n	419	0	30.7	30.72	194.07
13	3.9	Renal-right upper pole	n	558	0	30.7	30.72	194.07
14	1.7	Renal-left lower pole	n	180	0	28.5	38.21	437.23
15	2	Renal-right lower pole	n	233	0	28.4	48.42	299.61
16	2.2	Renal-right lower pole	n	299	0	28.4	48.42	299.61

Relative conspicuity of missed calculi between the CD-CT 5-mm ASIR-40 and the 5-mm LD-CT images reconstructed with FBP is scored on a 0–5 point scale (0 = not seen; 1 = much worse; 3 = equal; 4 = better; 5 = excellent)
